# *In silico* approach to screen compounds active against parasitic nematodes of major socio-economic importance

**DOI:** 10.1186/1471-2105-12-S13-S25

**Published:** 2011-11-30

**Authors:** Varun Khanna, Shoba Ranganathan

**Affiliations:** 1 Dept. of Chemistry and Biomolecular Sciences, Macquarie University, Sydney, Australia; 2 Dept. of Biochemistry, Yong Loo Lin School of Medicine, National University of Singapore, Singapore

## Abstract

**Background:**

Infections due to parasitic nematodes are common causes of morbidity and fatality around the world especially in developing nations. At present however, there are only three major classes of drugs for treating human nematode infections. Additionally the scientific knowledge on the mechanism of action and the reason for the resistance to these drugs is poorly understood. Commercial incentives to design drugs that are endemic to developing countries are limited therefore, virtual screening in academic settings can play a vital role is discovering novel drugs useful against neglected diseases. In this study we propose to build robust machine learning model to classify and screen compounds active against parasitic nematodes.

**Results:**

A set of compounds active against parasitic nematodes were collated from various literature sources including PubChem while the inactive set was derived from DrugBank database. The support vector machine (SVM) algorithm was used for model development, and stratified ten-fold cross validation was used to evaluate the performance of each classifier. The best results were obtained using the radial basis function kernel. The SVM method achieved an accuracy of 81.79% on an independent test set. Using the model developed above, we were able to indentify novel compounds with potential anthelmintic activity.

**Conclusion:**

In this study, we successfully present the SVM approach for predicting compounds active against parasitic nematodes which suggests the effectiveness of computational approaches for antiparasitic drug discovery. Although, the accuracy obtained is lower than the previously reported in a similar study but we believe that our model is more robust because we intentionally employed stringent criteria to select inactive dataset thus making it difficult for the model to classify compounds. The method presents an alternative approach to the existing traditional methods and may be useful for predicting hitherto novel anthelmintic compounds.

## Background

Besides malaria, infections due to nematodes are the leading cause of ailment to human beings. In particular, parasitic flatworms (cestodes and trematodes) and roundworms (nematodes) are a major cause of considerable suffering, mainly in children. According to a report by the World Health Organization (WHO) it is estimated that 2.9 billion people are infected with nematodes [1]. Therefore, to search for nematode specific targets is an active area under research. In Table 1, we present the list of successful biochemical targets and corresponding drug classes that are known to be active against those targets in helminths. With the availability of the completely sequenced nematode genomes, currently there is much interest to investigate drugs targeting their gene products.

**Table 1:** List of successful targets in helminths and corresponding drug class known to be active against those target.

S.No	Target	Biochemical class	BLAST score	Drug family
1.	Nicotinic acetylcholine receptor beta 1	Ion transport	E: 7e-27 62% identity with human NAch receptor beta 2	Cholinergic Agents
2.	Glutamate-gated chloride channel	Ion transport	E: e-137 54% similarity with human glutamate receptor	Macrolides
3.	Glutathione S-transferase	Transferases transferring alkyl or aryl groups	E: 6e-47 61% identity with human Mu isoform	Isoquinolines
4.	Tubulin beta	**−**	E: 0 96% similarity to human tubulin beta	Benzimidazoles
5.	Gamma-aminobutyric acid receptor	Chloride channel	**−**	Piperazines

At present however, only a couple of drugs are being used to control most worm infections in humans and animals. There are only three major classes of anthelmintic drugs available in the market. Benzimidazoles are broad spectrum anthelmintics and inhibit ß-tubulin resulting in impaired microtubule formation during cell division [2]. The benzimidazoles have greater affinity for tubulin in helminth cells than the tubulin found in the cells of mammals as first reported by Friedman and Plazer [3]. They found that fenbendazole was 250 times and mebendazole was 400 times more potent inhibitors of colchicine binding to *A. suum* embryonic tubulin than to mammalian tubulin and concluded that benzimidazoles clearly exhibit higher affinity to helminth tubulins. However, direct binding studies by Kohler and Bachmann [4] failed to find a significant change in benzimidazole affinity using mebendazole and intestinal *A. suum* tubulin. The authors surmised that differential pharmacokinetic behaviour of mebendazole could be responsible for the difference in drug susceptibility between host and parasite. Macrocyclic lactones form the second class of anthelmintics, interacting with a range of ion channels including glutamate-gated [5], γ-aminobutyric acid-gated [6] and acetylcholine-gated [7] chloride channels. Levamisole, pyrantel and morantel belong to the third class and bind to the nicotinic acetylcholine receptors causing muscle paralysis due to extended muscle contraction and spastic paralysis of the parasite [8]. Given the diversity in the chemical structures of these classes, predicting novel anthelmintics is a challenging task.

Nematodes infect the majority of the farm animals, and consequently, present a huge risk to livestock industry and exacerbate global food shortages. It is therefore not surprising that most of the anthelmintic drugs were originally developed to treat animal infections but were subsequently approved for human use with little or no modification. However, due to the disproportionate use of anthelmintics, currently the livestock industry is facing a very serious challenge with drug resistance in farm animals [9,10]. Furthermore, with a limited number of drugs being used, worm strains are able to develop drug resistance easily. In fact, there have also been reports of resistance for the present day anthelmintic drugs in humans [11]. Hence, there is an urgent need to discover novel safe and efficacious classes of anthelmintics with a new mode of action.

### Recent efforts in anthelmintic drug discovery

An excellent review on the current anthelmintics and existing research gaps that need to be addressed in order to discover novel anthelminthic drugs are summarized recently by Keiser and Utzinger [12]. Kaminsky *et al.*[13] reported a new class of synthetic anthelmintics, amino–acetonitrile derivatives (AADs) that are active against a variety of livestock pathogenic nematode species. The authors reported that the optimized AADs were able to eliminate fourth larval stages of *H. contortus*, *T. colubriformis* in sheep and *Cooperia oncophora*, *Ostertagia ostertagi* in cattle at a single oral dose of 20 mg racemate kg^-1^. The authors surmised that a unique group of nematode specific nAChR protein from *acr-23* gene is responsible for AAD efficacy. Hu *et al.*[14] have demonstrated that the mechanism of action of a novel anthelminthic drug, tribendimidine, approved recently in P.R. China. They concluded that tribendimidine is an L-subtype nAChR agonist, similar to levamisole pyrantel. The anthelminthic properties of cyclooctadepsipeptides have also been reported recently *in vitro* and *in vivo*[15,16]. Mefloquine is an antimalarial drug and has been used successfully for past four decades to treat prophylaxis of malaria. However, recent research revealed promising antischistosomal properties of mefloquine in *Schistosoma mansoni*- and *Schistosoma japonicum*-infected mouse models [17,18]. Ponce-Marrero *et al. *[19] introduced a novel approach for *in silico* design of new anthelmintic drugs using linear discriminant analysis to obtain a quantitative model that classified anthelmintic drug-like from non-anthelmintic compounds. The developed model correctly classified 88.18% of the compounds in external test set. The model was then used for virtual screening and several compounds from Merck Index and Negwer’s handbook were identified by the model as anthelmintic. Subsequently *in vivo* test were carried out to validate the predictions.

### Overview of the ligand-based virtual screening methods

Antiparasitic drugs historically have been discovered by experimental screening against intact parasites, but due to the enormity of the task and availability of better computational facilities there has been a shift towards computational screening. Computational screening (also known as virtual screening) has inherent advantage over traditional and even experimental high throughput screening (HTS) due to its massive parallel processing ability; millions of compounds per week can be tested. Virtual screening (VS) has been widely used to discover new leads by computationally identifying compounds with higher probability of strong binding affinity to the target protein. Successful studies have led to the identification of molecules either resembling the native ligands of a particular target or novel compounds [20,21]. VS methods can be classified into structure-based and ligand-based approaches based on the amount of structural and bioactivity data available. If the 3D structure of the receptor is known, a structure-based VS methods that can be used is high-throughput docking [22] but where the information on the receptor is scant, ligand-based methods [23] like similarity searching and machine learning techniques are commonly used. Docking involves a complex optimization task of finding the most favourable 3D binding conformation of the ligand to the receptor molecule. Being computationally intensive, docking is not suitable for very large virtual screening experiments. On the other hand, ligand-based methods are popular because they are computationally inexpensive and easy to use. Furthermore, the assumption that structurally similar molecules exhibit similar biological activity than dissimilar or less similar molecules is generally valid. Thus, ligand-based methods are increasingly playing an important role at the beginning of the drug discovery projects especially where little 3D information is available for the receptor. Particularly interesting are machine learning based approaches such as neural networks, genetic algorithms and support vector machines (SVM). SVM is a powerful classification technique that has found numerous applications in chemistry such as drug design, quantitative structure property prediction and chemical data mining. Many studies in the past have shown SVM to be one of the best methods for correctly classifying molecules [24-26]. Zernov *et al. *[24] used SVM and neural networks to predict the drug-likeness and agrochemical-likeness for large compound collections. They showed that for both kinds of data, SVM outperformed all neural networks under the same training conditions. Warmuth *et al.*[25] investigated a large collection of compounds to find those that bind to the target of interest in as few iterations of biochemical testing as possible. The authors compared various search strategies including maximum margin hyperplane, generated by SVM. They concluded that the strategies based on SVM clearly outperform the simpler ones. Similarly, Burbidge *et al. *[26] carried out a comparative study that involved prediction of the inhibition of dihydrofolate reductase by pyrimidines, using SVM, ANN and decision trees. They found that SVM outperformed the other methods, except in a manually capacity-controlled ANN, which required significantly longer training time. Nonetheless, ligand-based VS still remains an unproven approach in the discovery of antiparasitic medicines [27].

In this investigation, we have developed an *in silico* classification model using SVM to predict potential anthelmintic leads targeted towards parasitic nematodes. Our model has an estimated accuracy of ~82.0% for the test dataset. We have applied this model to a large public database to predict novel anthelmintic compounds and identified a set of 45 compounds, of which six are promising as potential therapeutic agents.

## Methods

### Preparation of the dataset

**Figure 1:**
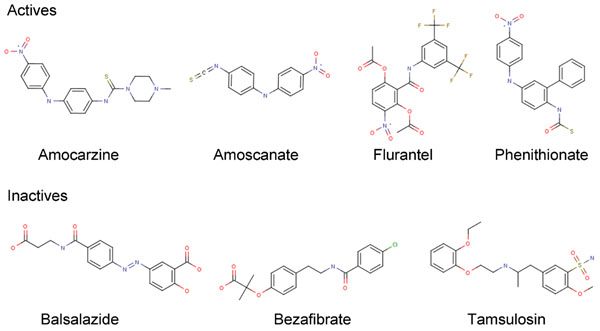
**Examples of active and inactive compounds used in this analysis.** The active compounds are collected from various literature sources and PubChem database while inactive compounds are adapted from DrugBank.

The quality of the data available largely determines the quality of any machine learning model [28]. Our primary dataset contains 295 unique compounds (148 actives and 147 inactives). The library of active molecules (compounds active against parasitic nematodes) was carefully collated from PubChem [29] and other literature sources [30-33]. For inactive compounds, we searched the DrugBank [34] database for similar molecules to the ones present in the active set with a Tanimoto cut-off range from 0.25 to 0.75. As a result, compounds from various pharmacological uses (anticancer, antibacterial, sedatives, antifungal) were collected into the inactive dataset. Since no true negatives (compounds without any anthelmintic activity) are reported in the literature, inactive compounds used in this study may possess residual anthelmintic activity. In Figure 1, we present representative active and inactive compounds used in this study for developing models. Further, the primary dataset was divided into training (80%) and testing sets (20%). The sampling was carried out at random and compounds in the test set were excluded from model development. In Table 2, we present the composition of the datasets used in this study. The training dataset was used for optimizing and training the SVM classifier [35] in order to predict compounds from an unseen test set. The training dataset contains 240 compounds (126 active and 114 inactive). The test dataset on the other hand was used for evaluating the performance of the SVM method and contains 55 compounds (22 active and 33 inactive). All the training set and test set compounds are available in Additional file 1. Based on our previous study, where we reported that the ChEMBL database [36] is quite diverse, contains many drug-like and interesting compounds, therefore, we used the ChEMBL database compounds for prediction set. Currently, the database holds over 650,000 compounds with calculated physicochemical properties (log P, molecular weight, Lipinski properties) and abstracted bioactivities (binding constant, pharmacology and ADMET data). We downloaded the ChEMBL dataset in SD format. After cleaning the dataset of any inconsistencies and inorganic structures, we removed the compounds with 0.8 or greater Tanimoto similarity to the compounds in primary dataset. Then we clustered the dataset to remove similar structures. Cluster centres were selected from each cluster while singletons were retained as such. For clustering, we employed the functional class substructural fingerprint as implemented in Pipeline Pilot software [37] with the Tanimoto cut-off value 0.7. This reduced our dataset to around 300,000 compounds. Finally, we randomly selected 10,000 compounds from ChEMBL dataset for descriptor calculation and further analysis.

**Table 2:** Composition of the datasets used in this study.

Dataset	Training set	Testing set	Total
Active	126	22	**148**
Inactive	114	33	**147**
**Total**	**240**	**55**	**295**
Prediction set (from ChEMBL)	−	−	10,000

### Defining scaffolds

**Figure 2:**
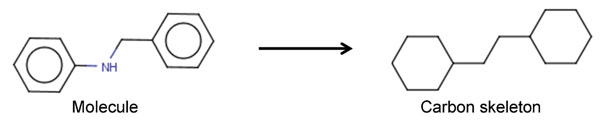
**Definition of the scaffold used in this study.** The scaffold is obtained by iteratively removing side chains and converting all the bonds to single bonds

In order to study the patterns in chemical compounds, it is important to decompose the molecules into fragments. There are a number of ways to fragment molecules as discussed elsewhere [38]. We describe below the specific method used in this study to obtain molecular scaffolds, where the term scaffold describes the core structure of the molecule (carbon skeleton). To obtain the carbon skeleton of the molecule, all the heavy atoms are represented as carbon and all bonds are converted to single bonds as shown in Figure 2.

### Descriptor calculation and selection

The determination of relevant features is an important step in any machine learning process [39]. Moreover, with hundreds of descriptors available it is essential to choose the best subset of descriptors because many of the descriptors are noisy and some are irrelevant to the target activity. Feature selection is the effective way to remove noisy or irrelevant descriptors and reduce the dimensionality of the feature space to avoid overfitting. This leads to simple and robust computational models with improved prediction accuracy.

There are two main approaches for feature selection in a supervised learning context. The first one is the filter approach [40]. It consists of selecting the best subset of features in an independent way, with *ad hoc* criteria. Filter methods are fast and can be easily implemented; however, there is no guarantee that the best subset of descriptors has been selected. The second method is the wrapper approach [41] which evaluates the performance of a predetermined learning algorithm and uses it as an evaluation criterion to select the optimum subset of features.

**Figure 3:**
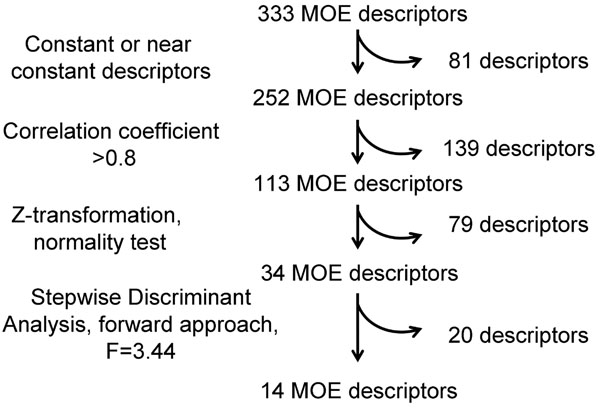
**Overall methodology adopted for descriptor selection.** Out of the total 333 MOE descriptors only 14 are used in this analysis.

The Molecular Operating Environment (MOE) [42] software was used for descriptor calculation. It calculates 333 descriptors, which are classified as one-dimensional (physicochemical properties), two-dimensional (topological) and three-dimensional (volume and surface area) descriptors. In Figure 3 we show the overall methodology adopted for descriptor calculation and selection. Due to the large number of descriptors available, we first filtered out constant and near constant descriptors (descriptors with <0.3 standard deviation). This resulted in the removal of 81 descriptors. Following this, we removed descriptors with a correlation coefficient greater than or equal to 0.8. The removal of correlated descriptors resulted in a set of 113 descriptors. Before performing univariate analysis, we normalized the dataset using the z-transformation. We then performed the normality test and those descriptors that passed the normality test were retained while the others were rejected. This reduced our previous set of 113 descriptors to 34 descriptors. For further selection of descriptors, we used the Stepwise Discriminant Analysis (SDA) [43] using a free data mining tool Tanagra [44]. SDA is often associated with discriminant analysis but in fact, it can be applied to various linear models such as linear SVM and logistic regression. However, it is not suitable for non-linear models such as multi-layer neural networks and nearest neighbours. We implemented SDA with both forward and backward elimination strategies. In the forward approach, at each step, all the variables are evaluated to determine which variables contribute maximum to the discrimination between the groups. Variables with significant contributions are included and the process starts again till there is no attribute to add to the model. In the backward approach, all the descriptors are included in the model and then, at each step, the descriptor that contributes least to the discrimination is eliminated, terminating when there is no descriptor to remove. For our problem, we found that the forward approach performs better than the backward elimination strategy. We used F statistics as the termination criterion, with a predefined threshold value of 3.44, where the F value for a descriptor indicates its statistical significance to discriminate between the positive and negative data groups. This resulted in the selection of final 14 descriptors out of 34. In Table 3 we present the final 14 descriptors used in this study.

**Table 3:** List of final 14 descriptors used in this analysis.

S.No.	Descriptor	Description
1.	AM1_HF	The heat of formation (kcal/mol)
2.	AM1_HOMO	The energy (eV) of the Highest Occupied Molecular Orbital
3.	ASA+	Water accessible surface area of all atoms with positive partial charge
4.	ASA-	Water accessible surface area of all atoms with negative partial charge
5.	ASA_P	Water accessible surface area of all polar
6.	E_ele	Electrostatic component of the potential energy.
7.	KierFlex	Kier molecular flexibility index
8	LogS	Log of the aqueous solubility (mol/L).
9.	Std_dim3	The square root of the third largest eigenvalue of the covariance matrix of the atomic coordinates.
10.	Vsurf_CP	
11.	Vsurf_CW2	Capacity factor
12.	Vsuf_D8	Hydrophobic volume
13.	Vsurf_EWmin	Lowest hydrophilic energy
14.	Vsurf_HB1	H-bond donor capacity

All the descriptors are derived from MOE software.

### SVM algorithm

The SVM algorithm was developed by Vapnik [45]. Recently, SVM has been applied to chemoinformatics, due to its robustness and ability to classify objects into two classes as a function of their features [46,47]. Many studies in the past have shown SVM to be one of the best methods for correctly classifying molecules [25,48]. A standard application of SVM involves defining two classes of objects, determining the set of features that distinguish these objects and use the trained SVM model to predict the classes of unknown data. Detailed accounts of the SVM methodology are present in literature [35,49]. Briefly, SVM is a new algorithm and is based on structural risk minimization principle from statistical learning theory. Each molecule to be classified by SVM is represented by a feature vector x_i_ (i=1,2…N) of *M* real numbers (descriptors) with the corresponding label y_i_ ε {+1,-1}, where y_i_ = -1 means inactive and y_i_ = +1 means active. To classify the data, the SVM attempts to find the optimal hyperplane {x ε R^m^: w.x +b =0} that best separates the input data into two classes in *M* dimensional space. The optimal hyperplane is defined in such a way that margin of separation between positive {x ε R^m^: w.x +b ≥ 0} and negative {x ε R^m^: w.x +b ≤ 0} examples is maximized with minimal error; where w is the normal vector of the hyperplane and b is the scalar. In other words, the optimal hyperplane passes through the “midpoint” between these sets. The decision function for new predictions on unseen examples is given in equation 1:(1)

where *K*(*xi*.*xj*) is the kernel function and the parameters are determined by maximizing the following equation 2:(2)

under the conditions (equation 3):(3)

The penalty constant *C* serves as a regularization parameter and represents the trade-off between minimizing the training set error and maximizing the margin. Higher number of support vectors is due to a small *C* and *vice versa*. If we use a very small *C* value, then almost all the samples would influence the model equally to build a decision boundary regardless of their position. As a result, virtually all the samples become support vectors. On the other hand, if we use a large *C* it may cause overfitting.

Since there are different types of kernels present (linear, polynomial, radial basis function, sigmoid) we explored various kernels for the efficacy of SVM prediction. From our analysis we note that radial basis function (RBF) kernel (equation 4) was found to be most effective (data not shown) therefore we have chosen the RBF kernel for further analysis.(4)

Two parameters *viz.*, *γ* which determines the capacity of the RBF kernel and the regularization parameter, *C* are required for optimization of SVM classifiers. To optimize the SVM parameters, *C* and *γ*, we carried out an extensive grid search to build accurate models. The resulting optimized parameters were *C* = 1.4 and *γ* = 0.43.

### Model validation

The prediction accuracy of the models developed was tested using ten-fold cross-validation technique. In a ten-fold cross-validation, the dataset was split into ten subsets of equal proportions. One of the subsets was used as the test set while the rest were used for training the classifier. The trained classifier was tested using the test set. This was repeated ten times using a different subset for testing and thus ensuring that every compound was used in prediction once.

### Performance measure

The prediction results from SVM were evaluated for the test dataset using the following statistical measures.

• TP, true positive – the number of correctly classified active compounds.

• TN, true negative – the number of correctly classified non-active compounds.

• FP, false positives – the number of incorrectly classified non-active compounds.

• FN, false negative – the number of incorrectly classified active compounds.

Using the variables above, a series of metrics were computed sensitivity (SN), specificity (SP), balanced accuracy (BA), F−measure and Matthews correlation coefficient (MCC).

The recall rate for the members of positive class (actives) is given by sensitivity, equation 5:(5)

Similarly, the recall rate for the members of the negative class (inactives) is given by the specificity, equation 6:(6)

Accuracy measures the ratio of correct predictions to the total number of classes evaluated. We calculated balanced accuracy which is given by the equation 7:(7)

Further, we calculated the F−measure, which is given by equation 8:(8)

Finally we calculated MCC from equation 9; the coefficient returns a value between +1 and -1. The higher the value of MCC, the better the classification result.(9)

## Results and discussion

The main aim of this study was to classify and predict novel compounds active against parasitic nematodes. The various molecular descriptors (333 in total) were calculated initially, using MOE [42]. After removing insignificant attributes (standard deviation ≤ 0.3) and applying a correlation test with a cutoff value of 0.8 we were able to reduce the total number of attributes to 113. Subsequently the SDA algorithm was applied and finally a set of 14 descriptors was selected for the development of classification model (details in Methods section).

The obtained model correctly classified 87.56% of the active compounds and 85.30% of the inactive compounds with the overall accuracy of 86.43% in the training set while 81.82% in the test set. The F−measure of the training and test sets are 86.52% and 79.17% respectively. Table 4 shows the result of the classification for the training and testing sets. All the predicted compounds can be found in Additional File 2.

**Table 4:** Performance measure of SVM classifier in training and test dataset.

Dataset	SN (%)	SP (%)	BA (%)	F-measure (%)	MCC
Training set	87.56	85.38	86.43	86.52	0.75
Test set	83.82	79.76	81.79	79.17	0.63

SN: sensitivity, SP: specificity, BA: balanced accuracy, MCC: Matthews correlation coefficient

The machine learning systems such as this could clearly reduce the cost involved in experimental methods involved in drug discovery pipeline. As the SVM algorithm has been effectively applied in various classification problems, we investigated the utility of SVM approach for the prediction of potential anthelmintic lead compounds. The accuracy of the model on the training dataset may indicate the effectiveness of a prediction model however; it may not be able to accurately show how the model will perform on novel compounds. Therefore, it is critical to test the model on an independent dataset, not used in training. In our case we trained and optimized the SVM classifier separately using the entire training set and evaluated the model on the test set. As shown in Table 4, the SVM model obtained an accuracy of 81.79% for the test set. On careful examination of our prediction result, we find that structural similarity of many false positives to the compounds in the active set is quite high, which may suggest a lower accuracy figure for the test set, due to our stringent threshold values. Further, we also note that a few false negatives are at the borderline and are thus classified as inactive by our model. To best of our knowledge, there are not many reported studies on the prediction of anthelmintic compounds therefore we were able to compare our results with only one study. We find that our results are comparable to that study. Marrero-Ponce *et al.*[19] used linear discriminant analysis to classify anthelmintic drug-like from non-anthelmintic compounds. The authors reported the accuracy of around 90.4 % in the training set while 88.2% in the test set which is slightly higher than ours. However, we believe our model is more robust because our selection criterion to pick inactive compounds was quite stringent. We selected molecules within the Tanimoto range of 0.25 to 0.75 of the compounds present in the active set which would make it relatively difficult to classify than if chosen randomly. The idea was to build a robust model that can classify compounds into separate groups even with structural similarity. Further, we surmise that since DrugBank covers most of the FDA approved drugs, the inclusion of DrugBank compounds in our inactive dataset would allow us to navigate to the unexplored regions of drug-like chemical space.

**Figure 4:**
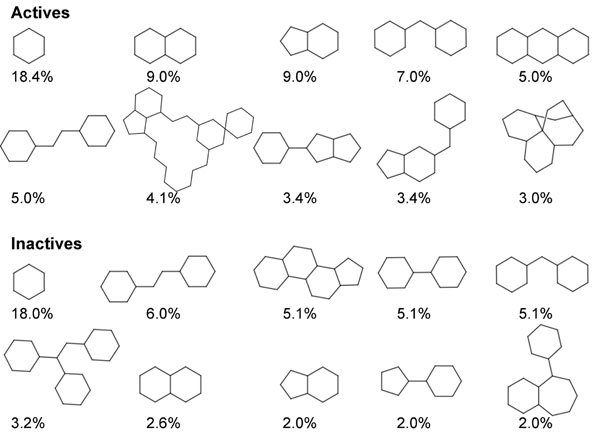
**Top ten scaffolds present in active and inactive dataset.** Inactive dataset is more diverse than active dataset. Five out of top ten scaffolds are shared in both the datasets.

The results obtained are particularly interesting from a clinical perspective. From our scaffold analysis we note that even though the size of both the dataset (active and inactive) is approximately same, the number of unique scaffolds found in the inactive set is almost twice the number of unique scaffolds found in active set. This clearly indicates that the inactive set is more diverse than the active set. The number of unique scaffolds, along with the relative percentage according to the total number of molecules present in the dataset is reported in Table 5. In Figure 4, we report the top ten molecular scaffolds in both the datasets. We note that, over 70.0% of the active compounds are represented by the top 10 scaffolds whereas only 51.1% of the inactive compounds are represented by the same number of scaffolds. This again suggests high scaffold diversity in inactive dataset. It should also be noted that five of the top ten scaffolds shown in Figure 4 are shared by both datasets.

**Table 5:** The number of unique scaffolds found in active and inactive sets along with the percentage relative to the dataset size.

Datasets	Size of the dataset	Non-redundant scaffolds	Percentage (relative to dataset size)
Actives	148	48	32.43%
Inactives	147	80	54.42%

**Figure 5:**
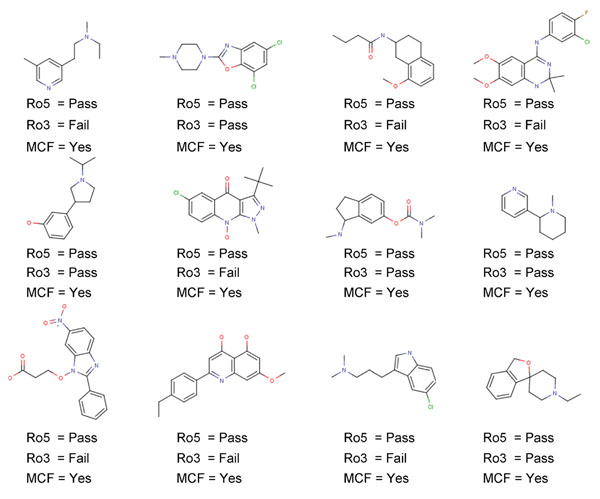
**Examples of the actives predicted in the prediction set derived from ChEMBL database.** All the molecules shown in the figure pass “rule of five” (Ro5) test and are medicinal chemist friendly (MCF). Further a few of them also pass lead-likeness “rule of three” (Ro3) test.

In the 45 predicted compounds, we note that piperazine-like substructures appear frequently suggesting that the nitrogen atom in the piperazine ring might be involved in binding to the receptor. Figure 5 shows an example set of predicted active compounds. Also, we note that many predicted compounds either contain benzimidazole scaffold or are derived from it *e.g.* in Figure 5, six compounds out of twelve are a derivative product of the benzimidazole scaffold. This shows the validity of the above method since the benzimidazole class of compounds are well recognized for anthelmintic activity [2]. Further, we searched the ChEMBL database for the binding affinity, assay type and target information of the identified compounds. We note that many predicted compounds bind to targets of interest in model organisms but experimental validation in the case of nematodes needs to be further carried out. Out of the total 45 predicted compounds six compounds are particularly interesting. Compound 3 with antiviral activity, compound 10 with inhibitory activity against *Ancylostoma ceylanicum* (a nematode), compound 12, compound 37 with antimicrobial activity against *Staphylococcus aureus*, compound 26 with activity to inhibit SARS-CoV 3CL protease enzyme and compound 40 with activity against Rhinovirus. In addition, there are compounds that bind to nicotinic acetylcholine receptor and tubulin β-1 chain in rats or humans. Since these two receptors are successful targets in nematodes, predicted compounds that bind to these targets can be used as leads to design novel compounds with high binding affinity to nematodes nicotinic acetylcholine and tubulin β-1 chain receptor.

## Conclusions

We were able to compile an extensive dataset of anthelmintic compounds for the development and validation of support vector machine model. We thoroughly tested the SVM approach for identifying the potential compounds with anthelmintic activity. From our results we conclude that SVM method is well suited for the prediction of anthelmintic (or antiparastic) compounds. We were also able to identify a number of interesting compounds with potential activity against parasitic nematodes however; experimental validation of the predicted compounds is needed.

## Authors' contributions

VK curated the datasets and conducted the analysis work, SR directed the study and both the authors prepared the manuscript.

## Conflict of interest

None declared.

## Supplementary Material

Additional file 1 **Table S1** Dataset used for training, testing and validation of the model. Click here for file

Additional file 2 **Table S2** Predicted compounds with AlogP, molecular weight and SMILES information. Click here for file
